# Mechanisms of autophagy and their implications in dermatological disorders

**DOI:** 10.3389/fimmu.2024.1486627

**Published:** 2024-11-04

**Authors:** Shenghao Xue, Yumeng Lin, Haoran Chen, Zhengyu Yang, Junting Zha, Xuan Jiang, Zhongyu Han, Ke Wang

**Affiliations:** ^1^ School of Medical and Life Sciences, Chengdu University of Traditional Chinese Medicine, Deyang Hospital Affiliated Hospital of Chengdu University of Traditional Chinese Medicine, Deyang, China; ^2^ Health Management Center, Nanjing Tongren Hospital, School of Medicine, Southeast University, Nanjing, China; ^3^ Chengdu Xinhua Hospital Affiliated to North Sichuan Medical College, Chengdu, China

**Keywords:** autophagy, psoriasis, systemic lupus erythematosus, vitiligo, atopic dermatitis, pemphigus, systemic sclerosis

## Abstract

Autophagy is a highly conserved cellular self-digestive process that underlies the maintenance of cellular homeostasis. Autophagy is classified into three types: macrophage, chaperone-mediated autophagy (CMA) and microphagy, which maintain cellular homeostasis through different mechanisms. Altered autophagy regulation affects the progression of various skin diseases, including psoriasis (PA), systemic lupus erythematosus (SLE), vitiligo, atopic dermatitis (AD), alopecia areata (AA) and systemic sclerosis (SSc). In this review, we review the existing literature focusing on three mechanisms of autophagy, namely macrophage, chaperone-mediated autophagy and microphagy, as well as the roles of autophagy in the above six dermatological disorders in order to aid in further studies in the future.

## 1 Introduction

Autophagy is an intracellular self-digestive process by which a cell breaks down and recycles damaged organelles, aberrant protein aggregates and other molecules from its interior to maintain normal cellular function and survival. Thus autophagy is considered to be fundamental to the maintenance of cellular homeostasis (1). Paradoxically, however, autophagy can also damage cells. For example, increased autophagy or defects in autophagy lead to an accumulation of autophagosomes, which can impair cellular function. Autophagy can also induce neuronal cell death in aggregated proteins, leading to neurodegenerative changes (2). Thus, dysregulation of autophagy causes disturbances in cellular homeostasis.

The skin is the largest organ of the body and is the first line of defense against many different environmental insults, including Ultraviolet (UV) radiation, pathogens, mechanical stresses and toxic chemicals. In addition, the skin contains a complex network of immune cells that reside in the tissues and are essential for host defense and tissue homeostasis (3). A large number of existing studies have shown that autophagy is activated and plays a role in maintaining skin homeostasis in skin cells, including keratinocytes, skin fibroblasts, melanocytes and immune cells (4). Thus, dysregulation of autophagy causes disturbances in skin homeostasis and also affects the development of various skin diseases.

In this review, we aim to describe the mechanisms of autophagy, the role of autophagy in skin cells, and the role of autophagy in skin diseases.

## 2 Molecular mechanisms of autophagy

Based on the mode of cargo transport, autophagy can be classified into three types, i.e. macrophage, CMA and microphage (5). Macrophages degrade cargo mainly through three steps: phagocyte nucleation, membrane expansion and autophagosome maturation (6). CMA degrades cargoes primarily through a chaperone-mediated process. During CMA, the chaperone heat shock homolog 70 (Hsc70) binds to substrate proteins to form a complex, which binds to lysosomal associated membrane protein 2 (LAMP2A) on the lysosomal membrane and enters the lysosomal lumen, and finally the substrate proteins are rapidly degraded by lysosomal proteases (7). In microphagy, it is mainly the lysosomes that take up the cytoplasmic component through membrane protrusion and invagination, leading to the degradation of that cytoplasmic component in the lysosomal lumen. However, microphagy of different cytoplasmic components has different molecular mechanisms (8).

Autophagy can also be divided into selective and non-selective autophagy. Both selective and non-selective autophagy proceed through a common overall mechanism that is mainly guided by a core autophagy mechanism consisting of autophagy-related proteins. Non-selective autophagy is usually induced by nutrient starvation (6). Selective autophagy is induced mainly by damaged organelles (mitochondria, lysosomes, endoplasmic reticulum, ribosomes), aggregation proteins and invading bacteria (9).

### 2.1 Molecular mechanisms of macrophagy

**Figure 1:**
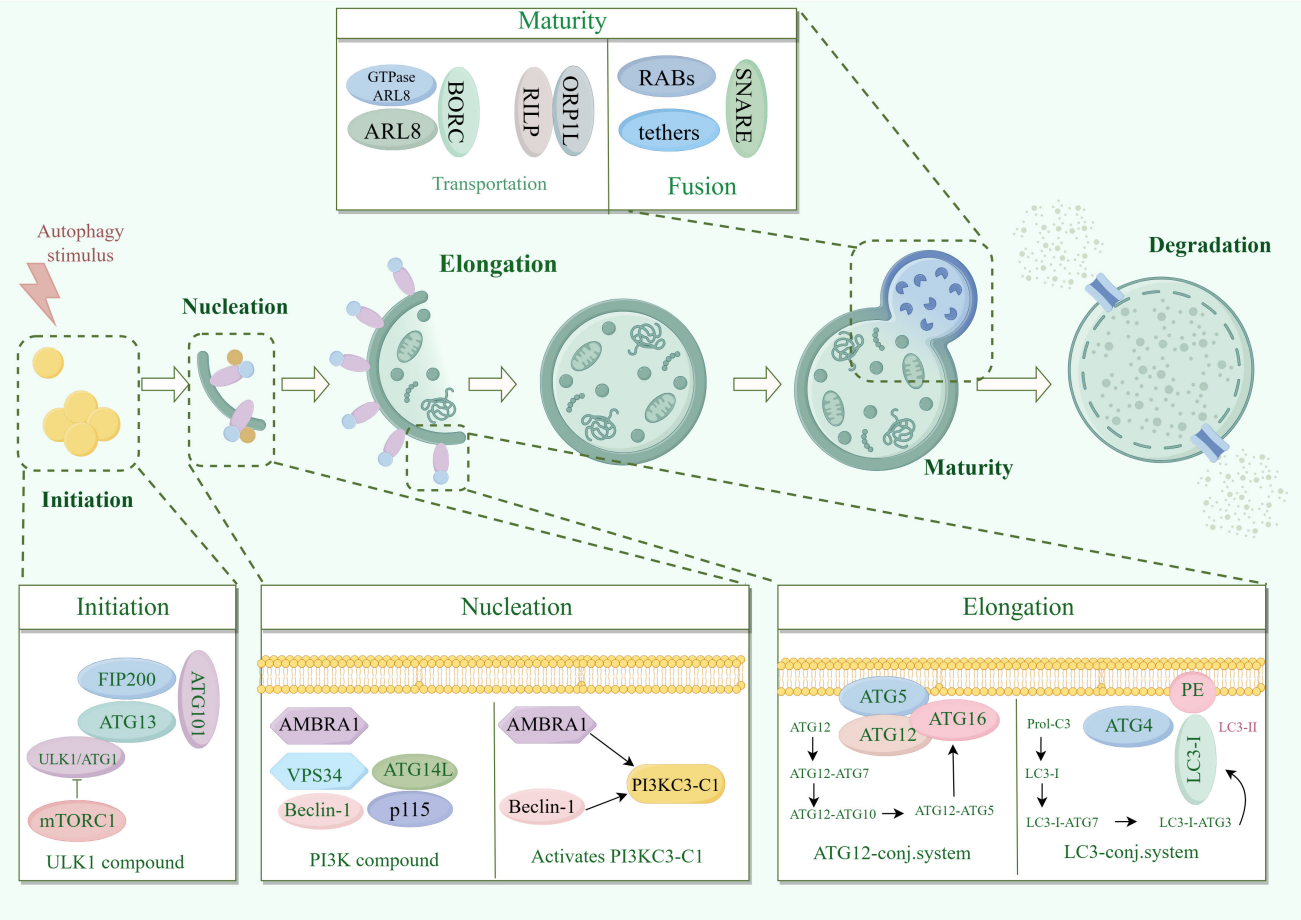
Molecular mechanisms of macrophagy. ATG13, autophagy-associated protein 13; AMBRA1, Autophagy and beclin 1 regulator 1 Gene; ARL8, ADP-ribosylation factor like protein 8; Beclin1, B-cell lymphoma-2 protein interaction center coiled-coil protein 1; FIP200, family of adhesion plaque kinase-interacting proteins; LC3, microtubule-associated protein light chain 3; mTORC1, mammalian target of rapamycin complex 1; PI3K, phosphatidylinositol 3-kinase; PI3KC3-C1, Type III phosphatidylinositol 3-kinase; RILP, Rab interacting lysosomal protein; SNARE, Soluble N-Ethyl cisbutylene diimide-sensitive fusion protein-linked protein receptor protein; ULK1, mammalian serine/threonine kinase; UVRAG, UV radiation resistance-associated gene; VPS34, type III phosphatidylinositol kinase.

The process of macrophage mainly involves: phagocyte nucleation, membrane expansion and autophagosome maturation (6) (**Figure 1**). Phagocyte nucleation occurs through three processes: stress signals acting on the target UNC51-like kinase-1 (ULK1) complex, phosphorylation of the phosphoinositide 3-kinase 3 (PI3K3) complex, and activation of the phosphatidylinositol-3-phosphate (PI3P). Through these three processes, phagocytes nucleate and generate membrane structural domains called ‘‘omegasomes’’ (6).

Stress signals include starvation, hypoxia, oxidative stress, protein aggregation, and endoplasmic reticulum stress. These stress signals act on the common target UNC-51-like kinase 1 (ULK1) complex (10). The ULK1 complex consists of ULK1 (yeast cells are autophagy-related gene 1 (ATG1)), ATG13, family of adhesion plaque kinase-interacting proteins (FIP200) and ATG101. In response to stress signaling, the ULK1 complex is called to the phagocytic assembly site (PAS) on the endoplasmic reticulum (ER) and activated by inhibiting mTOR (11, 12).

MTOR is present in two different protein complexes, mammalian target of rapamycin (mTORC1) and mTORC2, but only mTORC1 has a role in regulating autophagy (13). Activated ULK1 complex phosphorylates the PI3K3 complex. The PI3K3 complex consists of class III PI3K, type III phosphatidylinositol kinase (VPS34), B-cell lymphoma-2 protein interaction center coiled-coil protein 1 (Beclin-1), ATG14, autophagy and Beclin-1 regulator 1 (AMBRA1), and p115. ULK1 complex phosphorylated AMBRA1 is released from microtubules, binds to Beclin-1 and subsequently activates PI3KC3-C1 (14).

Activated PI3KC3-C1 is targeted to PAS by ATG14 through the interaction of ULK1 phosphorylation with ATG13 (15, 16). PI3KC3-C1 produces phosphatidylinositol-3-phosphate (PI3P) at PAS-activated endoplasmic reticulum characteristic structures (w vesicles). PI3P recruits the PI3P effector protein WD repeat structural domain phosphoinositide-interacting protein (WIPI2) and zinc-finger FYVE-containing structural domain protein 1 (DFCP1), which phagocytose cells to nucleate and generate membrane structural domains called omegasomes (6).

Most significantly associated with phagosomal membrane expansion is the Atg8 family (17). Atg8 has two domains. One domain is the C-terminal tail, which is covalently linked to the membrane. The other domain is globular, with a β-grasp fold, mediating a large number of protein-protein interactions. The function of Atg8 is to bind proteins regulated by autophagy and to localize them to sites of action on the membrane to promote membrane expansion (18).

Proteins are usually recruited to the phagosome via unique Atg8 interacting motifs (19). In addition to recruiting autophagy-related proteins to the phagosome, the Atg8 interaction motif also recruits autophagy-related regulators to the phagocyte, including Atg1 (ULK1), Atg13, and Atg4 and Atg7, which regulate the coupling and uncoupling of Atg8 with phosphatidylethanolamine (PE) (20).

In mammals, Atg8 is processed as microtubule-associated protein light chain 3 (LC3) at the C-terminus by Atg4 to generate LC3-I, which is subsequently activated by Atg7, which is then actively coupled to Atg3, and finally binds to PE to generate LC3-II, which promotes membrane expansion (21). In addition, Atg12 couples Atg5 as the E3 ligase of Atg8 via Atg10, which enhances the ability of Atg8 to attach to PE and promotes membrane expansion (11, 22). Atg5 is often present in non-covalent complexes with Atg16, which can immobilize Atg8-PE and Atg12-Atg5 couplers (23). Atg12 also calls Atg3 and promotes the coupling of Atg3 to Atg8 (24).

As phagocytosis expands and closes, the outer membrane of the nascent autophagosome gradually clears Atgs from the outer membrane and summons a mechanism for delivering lysosomes (6). Delivery of lysosomes includes kinesin motor-driven retrograde transport and microtubule kinesin dynamin-driven retrograde transport (25, 26). Paracrine transport is mediated by the BORC complex, which recruits the small GTPase ADP-ribosylation factor like protein 8 (ARL8) to facilitate ARL8-dependent coupling of the kinase motor (27).

It has been found that the RAB7 effector FYCO can also mediate cis transport by binding to LC3 and PtdIns3P (28). Retrograde transport is mediated by RAB7 and its effectors Rab interacting lysosomal protein (RILP) and ORP1L (27). Fusion of autophagosomes with lysosomes is accomplished by the synergistic action of RABs, tethers and soluble N-ethylmaleimide-sensitive factor attachment protein receptor (SNARE) complexes (29).

### 2.2 Molecular mechanisms of chaperone-mediated autophagy

**Figure 2:**
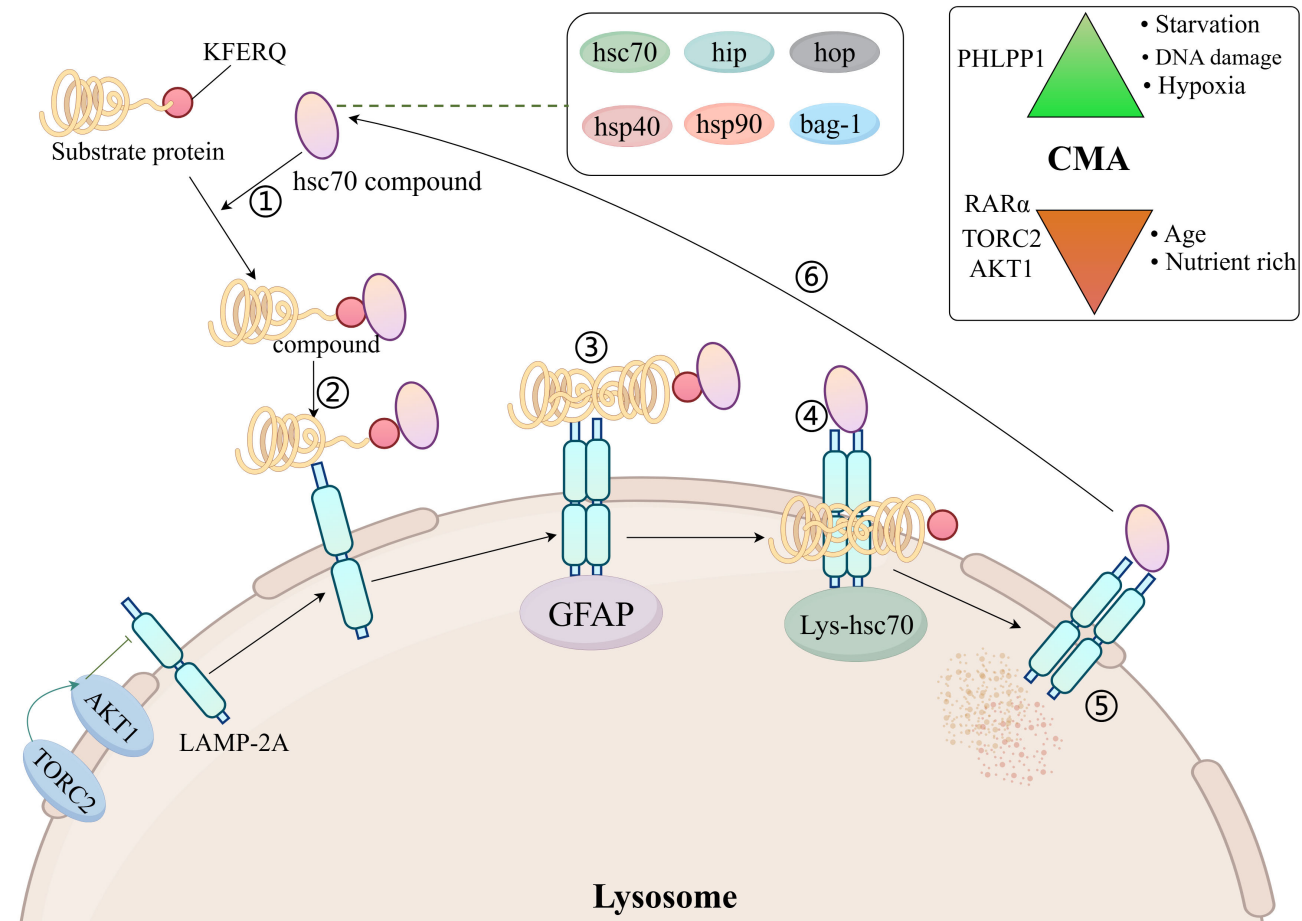
Molecular mechanisms of chaperone-mediated autophagy. AKT1, protein kinase Ba; LAMP 2A, lysosome-associated membrane protein 2A; hsc70, 70 kDa heat shock protein; TORC2, TOR complex 2; Top right: activators and inhibitors of CMA; GFAP, glial fibrillary acidic protein; PHLPP1, PH domain leucine-rich repeat-containing protein phosphatase 1; RAR, retinoic acid receptor; TORC2, TOR complex 2.

The chaperone-mediated autophagy process consists of six steps: Firstly, the formation of a complex between a 70 kDa heat shock protein (hsc70) and a region of the chaperone (hip, hop, hsp40, hsp90, and bag-1) recognizing the bound substrate protein (mainly KFERQ-related peptides); Secondly, the complex binds to lysosome-associated membrane protein 2A (LAMP 2A); Thirdly, the substrate protein unfolds on the lysosomal membrane in response to the hsc70-chaperone complex; Fourthly, the substrate protein enters the lysosome with the help of hsc70 in the lysosomal lumen; Fifthly, the substrate protein is degraded by the lysosome; Sixthly, the hsc70-chaperone complex is released from the lysosomal membrane and binds to the next substrate protein for recognition (30) (**Figure 2**).

For the time being, Hsc70 is the only chaperone that has been shown to bind directly to the substrate protein KFERQ motifs (7). Hsp40 increases the binding of hsc70 to substrate proteins by promoting the ATPase activity of hsc70 (31). Hip promotes binding of hsc70, hsp40 and substrate proteins (32). Hsp90 promotes folding of unfolded or misfolded proteins (33). Hop acts as a linker protein for hsc70 and hsp90 and will connect hsc70 to hsp90 (34). The isoforms regulating hsc70 consist of bag-1 (35). LAMP 2A is an isomer of LAMP 2. There are a total of three isomers of LAMP 2 (the remaining two being LAMP 2B and LAMP 2C), but only LAMP 2A is involved in CMA (36).

LAMP-2A is required for docking of the hSC70-substrate complex to the lysosome (37). Changes in LAMP-2A content in the lysosomal membrane dynamically regulate the rate of CMA (7). The amount of LAMP-2A on the lysosomal membrane is regulated in several ways: one part of LAMP-2A resides on the lysosomal membrane and the other part in the lysosomal lumen, and when the CMA is activated, LAMP2A from the lysosomal lumen enters the lysosomal membrane, causing an increase in the amount of LAMP-2A on the lysosomal membrane (38). When CMA is activated, LAMP-2A is stably degraded by lysosomal tissue proteinase A, causing a decrease in the amount of LAMP-2A in the lysosomal membrane (39).

LAMP-2A levels on lysosomal membranes are also negatively regulated by nuclear retinoid receptor-alpha (RARα) (40). It has been shown that the amount of LAMP-2A on lysosomal membranes is reduced in senescent fibroblasts and in the livers of aged rats, suggesting that ageing reduces the amount of LAMP-2A on lysosomal membranes (41). High-fat diet alters lysosomal membrane lipid composition, reduces LAMP-2A stability and inhibits CMA rates (42). In T cells, reactive oxygen species (ROS) production promotes increased LAMP-2A expression (43).

The Hsc70-chaperone complex is required for the unfolding of substrate proteins on the lysosomal membrane, and the unfolding process is mediated by glial fibrillary acidic protein (GFAP) (7, 44). After the substrate protein unfolds, it enters the lysosomal lumen from the lysosomal membrane and requires the translocation assistance of lysosomal luminal hsc70. Luminal hsc70 is necessary to complete the substrate protein translocation, and the lack of luminal hsc70 does not allow for CMA (30).

### 2.3 Molecular mechanisms of microphagy

**Figure 3:**
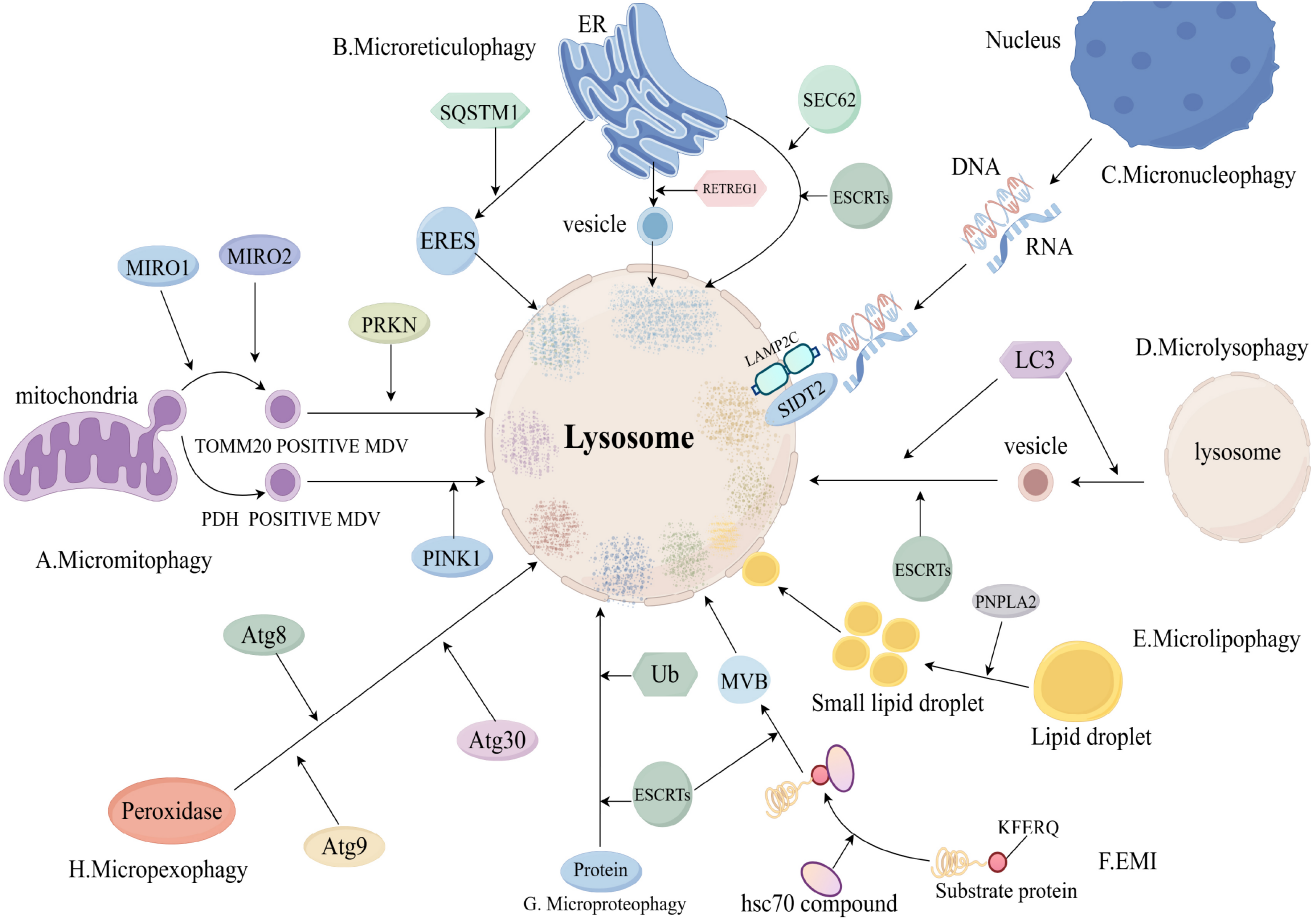
Molecular mechanisms of microphagy (Includes sections **A–H**). **(A)**, Micromitophagy molecular mechanism; **(B)**, Microreticulophagy molecular mechanism; **(C)**, Micronucleophagy molecular mechanism; **(D)**, Microlysophagy molecular mechanism; **(E)**, Microlipophagy molecular mechanism; **(F)**, Endosomal microautophagy (EMI) molecular mechanism; **(G)**, Microproteophagy molecular mechanism; **(H)**, Micropexophagy molecular mechanism. eres, endoplasmic reticulum exit site; mdv, mitochondria-derived vesicle; retregg1, reticulophagy regulator 1; mcss, membrane contact sites; mvb, multivesicular body; MIRO, Mitochondrial Rho GTPases; SQSTM1, Sequestosome 1; ub, ubiquitin.

The process of microphagy mainly involves the uptake of a cytoplasmic component by lysosomes through membrane protrusion and invagination, leading to the degradation of that cytoplasmic component in the lumen of the lysosome. However, microphagy of different cytoplasmic components has different molecular mechanisms (8) (**Figure 3**).

Micromitophagy(mitochondrial microphagy) is an important mitochondrial quality control mechanism to ensure mitochondrial homeostasis. The process of micromitophagy mainly includes: formation of mitochondria-derived vesicles (mdv), delivery of mdv to lysosomes, fusion of mdv with lysosomes, degradation of (45). Mdv was divided into mitochondrial outer membrane complex subunit 20 translocase (TOMM20)-positive mdv and pyruvate dehydrogenase (PDH)-positive mdv (8). The process of TOMM20-positive mdv formation consists of two parts: the microtubule-associated motor proteins mitochondrial Rho GTPase 1 (MIRO1) and MIRO2 and their binding partners emit mitochondrial protrusions, then, Dynamic protein 1-like gene (DNM1L) is recruited to mitochondria and catalyzes the breakage of mitochondrial protrusions (46).

TOMM20-positive mdv delivery to lysosomes is mediated by parkin RBR E3 ubiquitin protein ligase (PRKN) and Toll-interacting protein (TOLLIP) (47). PDH-positive mdv delivery to lysosomes is mediated by PINK1 and PRKN1 (48). Fusion and degradation of PDH-positive mdv with lysosomes is mediated by the SNARE complex (49).

There are three pathways of microreticulophagy (endoplasmic reticulum microphagy): ERES microautophagy, ER-to-lysosome-associated degradation (ERLAD), and piecemeal microreticulophagy (8). The ERES microautophagy pathway is dependent on the formation of endoplasmic reticulum exit sites (ERES) (50). ERES formation is dependent on LC3, solid solution 1 (SQSTM1) and ubiquitin (Ub) (51).

The ERLAD pathway consists of two components: formation of endoplasmic reticulum-derived vesicles mediated by reticulophagy regulator 1 (RETREG1) and LC3, and fusion degradation of endoplasmic reticulum-derived vesicles with endolysosomes mediated by SNARE complexes (52). Degradation of endoplasmic reticulum-derived vesicles by fusion with endolysosomes is mediated by the SNARE complex (52). The piecemeal microreticulophagy pathway is a direct delivery of endoplasmic reticulum into lysosomes, which is dependent on LC3, SEC62 and ESCRTs (53).

Micronucleophagy occurs in yeast cells through the interaction of Vac8 on the vesicle membrane and Nvj1 on the outer nuclear membrane (54). RNA/DNA microphagy is a specific type of micronucleophagy that delivers RNA/DNA to lysosomes for degradation via lysosome-associated membrane protein 2C (LAMP2C) and SID1 transmembrane family member 2 (SIDT2) (55, 56).

Microlysophagy has usually referred to macrophagy in past studies, but a recent study has shown that there is a new type of autophagy-lysosome-dependent lysosomal degradation in mammalian cells that does not depend on autophagosomes and belongs to the microphagy (57). The new microlysophagy is mediated by LC3 lipidation or ESCRTs (58, 59). LC3 is lipidated in the same manner as macrophage, and lysosomes promote the formation of lysosome-derived vesicles and fusion of vesicles with lysosomes by lipidating LC3 to degrade lysosomal membrane proteins (58).

Most autophagy-lysosome-dependent lipid degradation is accomplished by macrophages, but microlipophagy is also suggested in yeast cells and mammals (60, 61). The process of microlipophagy broadly involves three parts: Firstly, under starvation, large lipid droplets are converted to small lipid droplets by lipolysis of PNPLA2. Secondly, small lipid droplets contact with the lysosomal membrane to form membrane contact sites (MCSs). And last, small lipid droplets enter the lysosomal lumen through MCSs and are degraded (8).

Endosomal microautophagy (EMI) is the process by which cytoplasmic proteins carrying KFERQ-like motifs are delivered to late nuclear endosomes for degradation (62). The process of EMI is that hsc70 and its chaperones recognise and bind substrate proteins with KFERQ-like motifs to form a complex, which is transported to the late nuclear endosome for degradation (8).

Microproteophagy is the degradation of individual specific proteins by lysosomes independent of the occurrence of autophagosomes. Unlike EMI, microproteophagy does not degrade proteins containing specific motifs, such as KFERQ-like motifs. The exact mechanism of microproteophagy is not clear (63). Micropexophagy refers to the lysosomal degradation of degradative peroxidases that does not depend on the occurrence of autophagosomes. In yeast cells, micropexophagy is mediated by Atg8, Atg9 and Atg30 (64, 65).

## 3 The role of autophagy in skin cells

The skin is the largest organ in the human body and consists of three main layers: epidermis, dermis and subcutaneous tissue, and autophagy is the basis for the maintenance of homeostasis of skin cells (66). Keratinocytes are the outermost cells of the skin, accounting for 90% of the epidermal area, and become keratinocytes when they complete the process of differentiation and lose their nucleus, cytoplasm and organelles (67).

When the skin is damaged by environmental factors such as ultraviolet light, oxidative damage is caused to keratinocytes, and under oxidative stress, keratinocytes increase the expression of p62/SQSTM1, which induces autophagy and maintains the homeostasis of keratinocytes (68). When inflammation occurs in keratinocytes, granulinogen (PGRN) inhibits keratinocyte inflammation through the β-catenin signaling pathway. It has been shown that the expression of LC3II and Atg7 is suppressed when PGRN is specifically silenced, suggesting that autophagy is also involved in the anti-inflammatory action of keratinocytes (69). Autophagy plays an important role in keratinocyte differentiation, survival, senescence and resistance (4).

Fibroblasts are located in the dermis and are the cells in the dermis responsible for producing connective tissue and helping the skin to recover from damage (70). It has been shown that in senescent fibroblasts, the autophagic pathway is impaired, leading to deterioration of dermal integrity and skin fragility (71). In addition, fibroblasts can activate matrix metalloproteinase (MMP) and TGF-b signaling through autophagy to promote collagen production and alleviate skin ageing (72). Melanocytes are located in the basal layer of the epidermis of the skin and produce melanin. Melanin protects the subcutaneous tissue from ultraviolet radiation (73). But too much melanin in turn leads to hyperpigmentation of the skin. Autophagy is involved in the regulation of melanin production and degradation (74).

Autophagy plays different roles in different skin immune cells. Autophagy can help macrophages remove damaged proteins/organelles (75). Autophagy is key to the release of cytoplasmic granules from mast cells (76). Autophagy contributes to B cell differentiation and T cell homeostasis (77, 78). Autophagy is an important regulator of neutrophil function and neutrophil-mediated inflammation *in vivo (* 79). Autophagy enhances melanoma inhibition by NK cells (80).

## 4 The role of autophagy in skin disorders

### 4.1 Psoriasis

Psoriasis is a common chronic papulosquamous skin disease that is clinically characterized by chronic plaques that appear as orange-red patches covered with silvery scales on white skin and grey patches on black skin (81). Psoriasis has an uneven global geographic distribution, is more common in high-income countries and areas with ageing populations, and affects the health of more than 60 million people worldwide (82).

**Figure 4:**
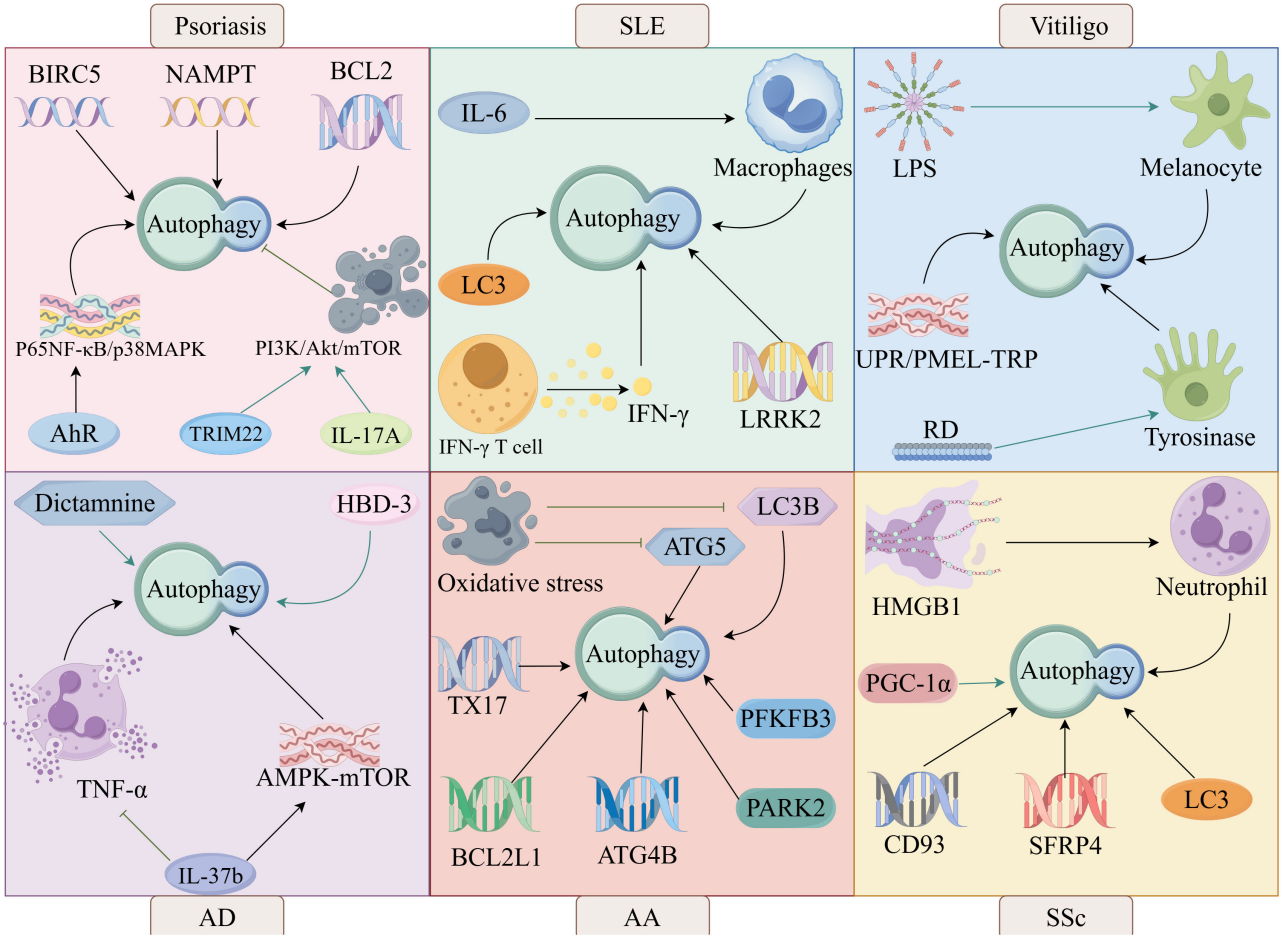
The autophagy in skin diseases, such as psoriasis, SLE, vitiligo, AD, AA, and SSc.

It has been shown that autophagy is involved in the pathogenesis of psoriasis. Aryl hydrocarbon receptor (AhR) regulates autophagy through the nuclear factor kappa-B (NF-κB)/mitogen-activated protein kinase (MAPK) signaling pathway leading to psoriasis, suggesting that AhR signaling and autophagy are involved in the pathogenesis of psoriasis (83). Autophagy-related genes BIRC5, NAMPT and BCL2 are potential biomarkers for early diagnosis of psoriasis vulgaris and are involved in psoriasis pathogenesis by regulating autophagy (84) (**Figure 4**).

Douroudis et al. performed a study of 241 unaffected controls (mean age 35.01 ± 13.60 years, 131 females) without a personal or family history of psoriasis and 299 patients with psoriasis (mean age 41.11 ± 13.87 years, age of onset 20.90 ± 8.16 years, 132 females) through the genetic validation and found that mutations in the autophagy regulatory gene ATG16L1 were associated with psoriasis (85). Satveer et al. validated the autophagy-related gene AP1S3 as a psoriasis gene through genetic screening of 85 patients recruited with psoriasis, and then they further experimented and found that AP1S3 contributes to psoriasis by disrupting autophagy in keratinocytes and up-regulating the production of IL-36 (86).

Inhibition of autophagy promotes psoriasis development. Aurora kinase A (AURKA) promotes psoriasis-associated inflammation by blocking autophagy-mediated inhibition of AIM2 inflammatory vesicles (87). TNF-α promotes psoriasis by causing impaired autophagy and lysosomal function (88). TRIM22 enhances keratinocyte inflammation and inhibits autophagy through activation of the PI3K/Akt/mTOR pathway, which in turn promotes psoriasis (89). In addition to this, IL-17A can also inhibit autophagy by activating the PI3K/Akt/mTOR pathway, which in turn promotes psoriasis-associated inflammation (90). IL-17A is a key pro-inflammatory cytokine in the pathogenesis of psoriasis, and anti-IL-17A is an effective treatment for psoriasis.

Currently, some clinical first-line drugs are used to treat psoriasis by activating autophagy through inhibition of the IL-17A. Suginumab, a fully human monoclonal antibody that selectively inhibits IL-17A, is approved for the treatment of psoriasis. James et al. found that plaque histopathology was reversed in the majority of patients with psoriasis after 12 weeks of treatment with sukinumab, suggesting that the activation of autophagy through inhibition of IL-17A facilitates plaque regression in patients (91). In addition to sukinumab, ezekizumab and brodalizumab are currently approved clinical first-line agents for the treatment of moderate to severe plaque psoriasis, and they also work by inhibiting IL-17A to activate autophagy (92). Of these, the slight difference is that brodalizumab acts as an IL-17 receptor (IL17R) inhibitor, while sukinumab and ezekizumab act as IL-17 inhibitors (93).

Celastrol, a natural compound extracted from the traditional Chinese medicine Lei Gong Teng, possesses anti-IL-17A properties. Celastrol inhibits the downstream signaling (NF-kB and MAPK pathways) of IL-17A by binding to it. which in turn induces autophagy to reduce psoriasis-associated inflammation (94). Fenofibrate is also an IL-17A inhibitor, similar to Celastrol, blocking the NF-kB and MAPK signaling pathways of IL-17A. Differently, fenofibrate also upregulates LC3 expression, inducing and enhancing autophagy (95).Lacin also inhibits IL-17A, which also directly inhibits the PI3K/Akt/mTOR pathway, thereby promoting keratinocyte differentiation and autophagy (96).

Inhibition of the PI3K/Akt/mTOR pathway is a key pathway for the activation of autophagy. In addition to laccasein, PSORI-CM02 (an herbal formula consisting of ginger rhizoma, paeonia lactiflora eryngii, paeonia lactiflora, smooth rhizoma, and paeonia lactiflora), LncRNA MEG3, YXJD (a herbal formula consisting of Salvia miltiorrhiza, Angelica sinensis, Radix Rehmanniae Praeparata, Japanese Snake Chicory, Scrophularia ningpoensis Hemsl, Spatholobi caulis, Smilax glabra Roxb, Paris multifoliage, Wrightia laevis, and Asian Plantain seeds) the mTOR inhibitors rapamycin, bittersweet, and avitamin A all play a role in alleviating psoriasis by activating autophagy through inhibition of the PI3K/Akt/mTOR pathway (97–101).

In addition, it has been shown that granulinogen (PGRN) induces autophagy through the β-catenin signaling pathway and can reduce psoriasis-associated inflammation (69). In summary, autophagy is involved in the pathogenesis of psoriasis, and activating autophagy is an effective means of treating psoriasis.

### 4.2 Systemic lupus erythematosus

SLE is a chronic multisystem autoimmune disease characterized by the presence of autoantibodies against nuclear antigens, immune complex deposition and chronic inflammation of target organs, with clinical symptoms including fatigue, lupus-specific rashes, mouth ulcers, alopecia, arthralgia and myalgia (102). SLE affects more than 3.4 million people globally, primarily affecting women, with a male-to-female patient ratio of approximately 1:9, and the mortality rate from SLE is much higher in low- and middle-income countries than in high-income countries (102).

It has been shown that autophagy is associated with the pathogenesis of SLE. IL-6 is involved in the pathogenesis of SLE by enhancing IL-6R-induced impairment of autophagic degradation in human macrophages (103). LC3-associated phagocytosis (LAP), an atypical defective form of autophagy, is involved in the pathogenesis of SLE (104). Increased IFN-γ T-cell autophagy found in patients with SLE (105). Increased autophagy in IFN-γ T cells leads to elevated plasma levels of IFN-γ, which in turn enhances disease activity in SLE. The autophagy-related gene leucine-rich repeat kinase 2 (LRRK2) is a susceptibility gene for SLE, and autophagy has beneficial effects on the pathogenesis of SLE (106).

Studies focusing on genome-wide associations have identified five autophagy-related genes associated with SLE susceptibility, including ATG5, ATG16L2, CDKN1B, DRAM1, and CLEC16A (107). In addition, Zhou et al. found that polymorphisms in the Prdm1-ATG5 intergenic region were also associated with SLE through a case-control association study of 1,745 individuals in the Chinese population, genotyping and meta-analysis of nine single nucleotide polymorphisms (108). Gao found that autophagy-related genes S100A8, MyD88 and NCR3 were associated with the pathogenesis of SLE by constructing a gene expression database and screening pivotal genes (109).

Some degree of inhibition of autophagy helps in the treatment of SLE. The escape and proliferation of autoreactive B cells is one of the characteristics of SLE, and the survival of autoreactive B cells is also dependent on autophagy; therefore, inhibition of autophagy will improve SLE (110). Belimumab, a monoclonal antibody that inhibits B lymphocyte-stimulating factor, is a first-line clinical treatment for SLE that inhibits B cells by suppressing autophagy, thereby reducing disease activity and severe flares in SLE (111). Belimumab is also highly tolerable and effective, which allows patients to reduce glucocorticoid use, thereby limiting the accumulation of damage (112).

Epratuzumab is a humanized IgG1 monoclonal antibody targeting CD22, which is used for the treatment of systemic lupus erythematosus (SLE) by inhibiting autophagy to block activation signals of the B-cell receptor and to promote the internalization of CD-22, thereby suppressing active B cells (113). Rituximab is a monoclonal antibody targeting CD20, which treats SLE by inhibiting immature, naïve and memory B cells, as well as B cells within the germinal centers, primarily through the inhibition of autophagy (114).

It has been shown that miR-125b inhibits autophagy by targeting the ultraviolet radiation resistance-associated gene (UVRAG), which in turn ameliorates SLE, suggesting that UVRAG is a potential target for the treatment of SLE (115). Autophagy causes an imbalance in Th17/Treg immunity in SLE, but chloroquine inhibits autophagy, rebalances Th17/Treg immunity, and improves SLE (116). P140 peptide significantly ameliorates SLE, which exerts this effect by inhibiting autophagy through binding to HSC70 protein (117).

Increased macrophage apoptosis in SLE patients (118). Macrophages are important immune cells that remove necrotic material, and increased macrophage apoptosis promotes the development of SLE (119). It has been reported that the Notch1-Hes-1 axis controls TLR7-induced macrophage autophagic death by regulating P62 (120), Suggests that inhibiting Notch1-Hes-1 to suppress macrophage autophagy is a potential target for the treatment of SLE. Lupus nephritis (LN) is triggered by the accumulation of SLE in the kidney, and vitamin D can treat LN by reducing autophagy to protect podocytes (121).

However, some degree of induction of autophagy can also be helpful in the treatment of SLE.SGLT2 inhibitors attenuate podocyte damage in the LN by reducing inflammation and enhancing autophagy, which in turn treats the LN (122). Honokiol (HNK), a major anti-inflammatory bioactive compound in Magnolia, reduce NF-κB/NLRP3 inflammatory vesicles by activating the SIRT1 autophagy axis and negatively regulating T-cell function, which in turn treats LN (123). In summary, autophagy is involved in the pathogenesis of SLE and autophagy is a potential target for the treatment of SLE.

### 4.3 Vitiligo

Vitiligo is an acquired chronic skin pigmentation disorder characterized by the destruction of functional melanocytes in the epidermis, and is clinically characterized by the appearance of creamy-white, non-scaly patches of skin with distinctive edges (124). Vitiligo is the most common skin pigmentation disorder, with a prevalence of 0.06%-2.28% in the general population and 0%-2.16% in children, with adults and children of both sexes equally affected (125).

Autophagy is involved in the pathogenesis of vitiligo. It has been shown that lipopolysaccharide (LPS) can be involved in the pathogenesis of vitiligo by inhibiting melanin formation in vitiligo melanocytes through the activation of melanocyte autophagy and down-regulation of melanin synthesis-related protein expression (126). A new autophagy axis, the unfolded protein response (UPR)/pre-melanosomal protein (PMEL)-transient receptor potential (TRP) autophagy axis, is involved in the pathogenesis of vitiligo, which involves activated UPR systems, impaired PMEL accumulation, and autophagic imbalance between mitochondria and lysosomes via TRP channels (127).

Rhododendrol (RD) is a naturally occurring phenolic compound that induces albinism in mice by activating the tyrosinase autophagy pathway (128), suggests that RD can participate in the pathogenesis of vitiligo by modulating autophagy. Furthermore, Luo et al. identified five mitochondrial autophagy-associated DEGs (GABARAPL2, SP1, USP8, RELA, and TBC1D17) by hybridizing vitiligo differentially expressed genes (DEGs) with mitochondrial autophagy-associated genes, suggesting that mitochondrial autophagy may promote vitiligo by activating immune infiltration (129). However, it has been shown that capsaicin in combination with mesenchymal stem cells (MSCs) can ameliorate mitochondrial autophagy abnormalities through inhibition of the HSP70/TLR4/mTOR/FAK signaling axis (130).

Zhao et al. verified potential genes associated with vitiligo autophagy by bioinformatics analysis and experimental tests and found that autophagy-related genes CCL2, RB1CC1, TP53 and ATG9A would have an impact on vitiligo development (131). Yang et al. identified differentially expressed autophagy-associated genes (DEARGs) in vitiligo by RNA sequencing and found that 39 DEARGs were present in vitiligo lesions, suggesting that autophagy is involved in vitiligo pathogenesis by participating in multiple pathways and biological functions (132).

To some extent, induction of autophagy helps vitiligo treatment. It has been shown that activation of the HSF1-ATG5/12 autophagy axis protects melanocytes from surviving oxidative stress, thereby improving vitiligo (133). Calcipotriol can protect melanocytes from oxidative damage in vitiligo by activating mitochondrial autophagy, thereby improving vitiligo (134). Lycium barbarum polysaccharide (LBP) can activate autophagy and promote melanocyte proliferation by activating the Nrf2/p62 signaling pathway, thus anti-vitiligo (135).

In addition to this, it has been suggested that impairment of the Nrf2-p62 signaling pathway leads to dysregulation of autophagy and increased sensitivity of melanocytes to oxidative stress (136), suggests that the Nrf2-p62 signaling pathway is a potential target for the treatment of vitiligo. Under oxidative stress conditions, TRPM2 inhibits autophagy by suppressing the coupling of Atg12 and Atg5 and promotes the secretion of CXCL16, a chemokine with melanocyte-killing effects. Therefore, inhibiting TRPM2 to induce autophagy can improve vitiligo (137).

However, some degree of inhibition of autophagy can also treat vitiligo. Janus kinase (JAK) inhibitors can suppress autophagy by inhibiting the JAK-signal transducers and activators of transcription (STAT) pathway (138). JAK inhibitors are now used as first-line clinical agents for the treatment of many immune-related diseases (139). Ruxolitinib cream is an inhibitor that selectively targets JAK1 and JAK2 (140). In two phase 3 trials, 674 vitiligo patients from North America and Europe were randomly assigned in a 2:1 ratio to apply 1.5% ruxolitinib cream or vehicle control to all areas of vitiligo on the face and body twice a day for 24 weeks, after which all patients were allowed to use 1.5% ruxolitinib cream until week 52. At week 52, application of ruxolitinib cream resulted in greater repigmentation of vitiligo lesions than vehicle control, suggesting that Ruxolitinib cream, as a first-line clinical agent, has a significant effect on the treatment of vitiligo (141).

Tofacitinib, a selective JAK1 and JAK3 inhibitor, is effective against vitiligo when administered orally or topically (142). Brepocitinib is an oral JAK1 inhibitor for the treatment of moderate to severe plaque psoriasis (143). Ritlecitinib is an oral JAK3 and tyrosine kinase inhibitor which is used in the treatment of active non-segmental vitiligo (144). In summary, autophagy is involved in the pathogenesis of vitiligo, and autophagy is a potential target for the treatment of vitiligo.

### 4.4 Atopic dermatitis

AD is a generalized inflammatory skin disease, usually beginning in childhood, characterized by impaired epidermal barrier function and an over-activated immune system, with clinical features including recurrent, itchy, limited eczema, often with seasonal fluctuations. The prevalence of AD is approximately 10 per cent in adults and 15-20 per cent in children, with prevalence rates varying according to gender and ethnicity (145).

Autophagy is involved in the process of AD and stimulation of autophagy is a therapeutic option for AD. Stefan et al. analyzed the whole transcriptome of the skin of AD patients and healthy individuals by applying RNA sequencing and found that the expression of autophagy-related genes ULK1, ATG4, and ATG16L2 was increased in AD patients (146). Kim et al. found elevated levels of genes expressing the autophagy-related proteins ATG5, ATG7, LC3B, and p62 in the epithelium of patients with AD by examining the levels of key ATG proteins in human skin specimens as well as in primary human epidermal keratinocytes exposed to inflammatory stimuli *in vitro (* 147). This is consistent with the findings of Ge et al. who observed reduced LC3 levels and elevated p62 levels in the epithelium of AD patients and AD mouse models (148).

IL-4 and IL-13 mediate epidermal dysfunction in AD, and it has been shown that human β-defensin-3 (hBD-3) attenuates the impaired epidermal function caused by IL-4 and IL-13 through activation of the autophagy and aryl hydrocarbon receptor (AhR) signaling pathways, thereby treating AD (148). Cortex Dictamni is a traditional Chinese medicine widely used in the treatment of dermatitis, and Dictamnine is one of its main ingredients, and it has been suggested that Dictamnine can improve AD by inhibiting M1 macrophage polarization and promoting autophagy (149).

Moisturizing agents with autophagy-stimulating effects help skin barrier restoration and inflammation control for the treatment of AD (150). The scaffolding protein p62/SQSTM1 is an autophagy receptor (151), WLJP-025p is a polysaccharide extracted from the traditional Chinese medicine Honeysuckle, and it has been shown that WLJP-025p can stimulate autophagy by up-regulating the expression of p62, and promote the ubiquitination and degradation of NLRP3, thereby improving AD (152). Prolonged exposure to TNF-α impairs autophagy and lysosomal function in keratinocytes and can promote chronicity of AD (88), However, IL-37b can inhibit TNF-α to improve autophagy and thus improve AD (153). IL-37b also improves AD by modulating the AMPK-mTOR autophagy signaling pathway and gut bacteria (153).

However, some recent studies suggest that some degree of autophagy inhibition may also have a helpful effect in the treatment of AD. JAK inhibitors can treat AD by inhibiting autophagy (154). Delgocitinib is a JAK1, JAK2, JAK3, and Tyk2 (pan-JAK) inhibitor, and 0.5% Delgocitinib Ointment is used to treat AD in adults and 0.25% Delgocitinib Ointment is used to treat AD in children (154). Tofacitinib 5 mg orally twice daily in adults can be used to treat moderate to severe AD (155). In addition, topical Tofacitinib can be used to treat mild to moderate AD (156).

Although no studies have shown that oral Ruxolitinib can be used to treat AD, topical Ruxolitinib formulations can be used to treat mild to moderate AD In a phase II trial, 307 adult AD patients were randomly assigned to receive an average of 8 weeks of Ruxolitinib ointment treatment (1.5% twice daily [BID], 1.5% daily once daily [QD], 0.5% QD, and 0.15% QD), and at week 8, results showed that 1.5% BID provided the greatest improvement in AD, suggesting that Ruxolitinib ointment provides rapid and sustained improvement in AD (157). Baricitinib, an oral selective inhibitor of JAK1 and JAK2, is used to treat moderate to severe AD in adults at a dose of 2 mg QD (158). Abrocitinib, an oral JAK1 selective inhibitor, can be used to treat moderate to severe AD in adults at a dose of 100 mg QD (159).

Lack of macrophage autophagy leads to accumulation of the transcription factor CCAAT/enhancer binding protein beta (CEBPB), accumulation of CEBPB upregulates the expression of SOCS1 and SOCS3, and increased expression of SOCS1 and SOCS3 ameliorates AD by inhibiting M2 macrophage polarization (160). Taken together, autophagy is a potential target for the treatment of AD.

### 4.5 Alopecia areata

AA is a common, inflammatory, non-scarring form of alopecia areata whose common clinical manifestation is the presence of well-defined inflammatory, non-scarring plaques on the scalp, characterized by exclamation point hairs, dystrophic hairs and yellow spots (161). The prevalence of AA is 0.1-0.2 per cent, with the majority of patients younger than 30 years of age, and AA affects both sexes equally (162).

Autophagy has been shown to be involved in the regulation of AA. Genome-wide association studies of AA have shown that susceptibility to AA is not only associated with two autophagy-related pathways, PARK2 and PFKFB3, but also with two autophagy-related genes, TX17 and BCL2L1 (163). In addition to this, copy number variation in the autophagy-related gene ATG4B was present in patients with AA (164). Regina et al. identified the autophagy-related genes STX17 and CLEC16A as susceptibility loci for AA in a meta-analysis of 3,253 cases and 7,543 controls by combining data from two genome-wide association studies (GWAS) (165).

It was shown that impaired autophagy exacerbated AA in the C3H/HeJ mouse model, whereas pharmacological induction of autophagy restored autophagic activity and reduced associated inflammation in AA skin, suggesting that autophagy is implicated in the pathogenesis of AA (166). Under conditions of oxidative stress, autophagy-associated factors ATG5 and LC3B are reduced in the hair matrix, suggesting that autophagy in the hair follicle is reduced during the development of AA (167).

However, cysteine (an amino acid essential for hair growth) can protect hair follicles from oxidative stress damage by regulating autophagy, thereby improving AA (168). In addition to this, PTEN-induced kinase 1 (PINK1)-mediated mitochondrial autophagy can ameliorate AA by inhibiting NLRP3 inflammatory vesicles, suggesting that mitochondrial autophagy may be a potential target for the treatment of AA (169).

JAK inhibitors can treat AA by inhibiting autophagy (170). Baricitinib is a JAK inhibitor for the treatment of adult patients with severe AA. In two phase 3 trials, 654 patients with AA and 546 patients with AA were randomized in a 3:2:2 ratio to receive once-daily doses of 4 mg of baricitinib, 2 mg of baricitinib or placebo for 36 weeks. At week 36, hair regrowth was superior in patients with barectinib at a dose of 4 mg to those with barectinib at a dose of 2 mg to those with placebo (171). Ritlecitinib is a JAK inhibitor approved for the treatment of patients aged 12 years and older with severe AA (172). Ritlecitinib may be the most appropriate treatment option for patients with AA who are candidates for systemic therapy (173). Baricitinib and ritlecitinib are both oral JAK inhibitors, and topical JAK inhibitors (ruxolitinib cream, delgocitinib ointment, and tofacitinib ointment) are ineffective in moderate to severe AA (170).

Some degree of induction of autophagy also helps in the treatment of AA. Min et al. found that the metabolites α-ketoglutarate (α-KG) and α-ketobutyric acid (α-KB), as well as the prescription drugs rapamycin and metformin, which affect mTOR and AMPK signaling, promote hair growth in resting follicles through activation of autophagy to treat AA (174). This is consistent with the findings of Chiara et al. who found that inhibition of autophagy leads to apoptosis-driven degeneration of hair follicle isolates (175). Furthermore, Cai et al. found that activation of autophagy promotes differentiation of hair follicle stem cells (176).

Quercetin, an ingredient of traditional Chinese medicine, is a bioflavonoid with anti-inflammatory properties. Tongyu et al. found that quercetin could activate autophagy by activating the NF-κB signaling pathway, thereby reducing the production of pro-inflammatory cytokines, through subcutaneous injection of quercetin into mice suffering from spontaneous AA, suggesting that quercetin could alleviate AA by activating autophagy (177). Ginger is the fresh rhizome of ginger, an herb in the ginger family. Abbas found that ginger can act to alleviate AA by activating autophagy to inhibit impaired oxidative homeostasis in AA patients (178).

Some studies have shown that adequate dietary protein and eating breakfast early can prevent autophagy dysregulation in hair follicles, thus preventing AA (179). In summary, autophagy is involved in the pathogenesis of AA and autophagy is a potential target for the treatment of AA.

### 4.6 Systemic sclerosis

SSc is an immune-mediated disease characterized by fibrosis of the skin and internal organs as well as vasculopathy, with a wide variety of clinical manifestations, notably Raynaud’s phenomenon, gastro-esophageal reflux, skin tightness and pruritus (180). The prevalence of SSc ranges from 7.2 to 44.3 cases per 100,000 adults, with females being 3.8 to 15 times more likely than males, and the mortality rate for SSc is one of the highest of all connective tissue diseases (181).

Autophagy has been shown to be involved in the pathogenesis of SSc.PGC-1α promotes TGFβ-induced fibroblast activation and tissue fibrosis by facilitating autophagy, thus participating in the pathogenesis of SSc (182). It has been found that blood levels of HMGB1 are elevated in patients with SSc and that HMGB1 maintains autophagy-associated activation of neutrophils, suggesting that neutrophil autophagy is involved in vasculopathy in SSc (183).

Higher LC3 expression and increased autophagy were found in skin specimens from SSc patients compared to healthy controls (184), This finding is consistent with the findings of Tatsuhiko Mori et al., who found a significant increase in the number of positive points for LC3 in bleomycin-induced scleroderma skin of mice, suggesting that autophagy activation contributes to the pathogenesis of SSc (185). Mori et al. also found that autophagy activation was also present in lung fibroblasts from SSc patients by looking at human samples (185). Zehender et al. found that autophagy was upregulated in the fibrotic skin of SSc patients, with increased expression levels of the autophagy-related genes LC3, Beclin1, and ATG7, and decreased expression levels of p62 (186). Notably, Zhou et al. found the involvement of autophagy in the pathogenesis of SSc by genomics and epigenomics (187).

In addition to this, Veronica et al. also found an increase in autophagic flux in SSc dermal fibroblasts (188), Liu Chen et al. also found higher expression levels of autophagy-related genes CD93 and SFRP4 in patients with SSc (189), These studies have shown a close link between autophagy activation and the pathogenesis of SSc.

Remedios et al. studied SSc characteristics in foveal protein-1-deficient mice and found that inhibition of autophagy prevented fibrosis in SSc patients, leading to the treatment of SSc (190), This finding is consistent with the findings of Zhu Ke et al. and Liu Chaofan et al. Zhu Ke et al. found that 2-methoxyestradiol (2-ME) could prevent fibroblast collagen synthesis and endosomal transformation in SSc by inhibiting autophagy, thereby improving SSc (191), Chaofan Liu et al. found that exosomal miR-126-3p can inhibit autophagy by regulating the SLC7A5/mTOR signaling pathway in human umbilical vein endothelial cells (HUVECs), thereby preventing SSc vascular injury (192). Zehender et al. found that inhibition of autophagy makes human fibroblasts less sensitive to the pro-fibrotic effects of TGF-β, thereby inhibiting TGFβ-induced fibroblast activation and ameliorating dermal fibrosis (186).

JAK inhibitors can block the TGF-β-mediated STAT protein activation pathway by inhibiting autophagy, thereby inhibiting fibrosis in the skin of SSc patients and achieving the treatment of SSc (193). Nintedanib is a JAK inhibitor used to treat SSc. In a double-blind trial, 576 patients were randomly assigned in a 1:1 ratio to receive either 150 mg of Nintedanib (taken orally twice a day) or placebo for 52 weeks, and at week 52, patients taking oral Nintedanib had less fibrosis and inflammation than those taking oral placebo (194). Tofacitinib is currently the most commonly used JAK inhibitor for the treatment of SSc patients. In one of the earliest cases of SSc treatment with tofacitinib, the patient was a young patient who had failed mycophenolate mofetil therapy, and the patient experienced significant improvement in systemic symptoms by taking tofacitinib 5 mg twice daily for several months (195).

Dang Gui Xie Blood Tablet (DHP) is a traditional Chinese herbal formula used for the treatment of SSc. DHP inhibits autophagy by inhibiting the TGF-β1 signaling pathway, thereby improving SSc skin inflammation, fibrosis and vascular lesions (196). A Chinese herbal formula called “Yiqi and Blood Formula” may also inhibit autophagy by inhibiting the TGF-β1 signaling pathway, thus exerting an anti SSc fibrosis effect (197). Comfreyin, extracted from the rhizome of the traditional Chinese herb comfrey, can inhibit autophagy by inhibiting the NF-κB signaling pathway, thereby attenuating the inflammatory response in SSc (198).

Some studies have shown that some induction of autophagy can also be helpful in the treatment of SSc. Zhou et al. found that activation of autophagy by inhibiting the PI3K/Akt/mTOR signaling pathway reduced the production of the fibrotic cytokines connective tissue growth factor (CTGF) and collagen I in SSc fibroblasts (199). This is consistent with the findings of Liang et al., who found that activation of autophagy through inhibition of the PI3K/Akt/mTOR signaling pathway could produce excellent inhibition of fibrosis in the skin in the SSc mouse model (200). In summary, autophagy is involved in the pathogenesis of SSc, and autophagy is a potential target for the treatment of SSc.

## 5 Targeting autophagy-related proteins or pathways for therapy

Autophagy is involved in and regulates the pathogenesis of psoriasis, SLE, vitiligo, AD, AA, and SSc, and targeting autophagy-associated proteins or pathways can treat these diseases (**Table 1**). Some studies found that the expression of LC3, an autophagy-related protein, decreased or even disappeared in psoriasis lesional epidermis, suggesting that autophagy is impaired in psoriasis, suggesting that targeting to increase the expression of LC3 could be a therapeutic option for psoriasis (201). However, several other studies have shown that the expression of LC3 and other autophagy-related proteins (Beclin1, ATG5 and ATG7) is also elevated in psoriasis, which is hypothesized to be a possible compensatory mechanism for impaired autophagy (147, 202–204).

**Table 1:** Promoting/inhibiting autophagy for the treatment of skin diseases.

Skin diseases	Treatment	Study subject	Mechanism	References
Psoriasis	Celastrol	HEK-Blue cell Female C57BL/6 mice	Anti-IL-17.	(94)
	Fenofibrate	Female C57BL/6 mice HaCaT cell	Anti-IL-17.	(95)
	Fexofenone	Psoriasis patients Female C57BL/6 mice Female BALB/c mice	Anti-IL-17.	(96)
	PGRN	Psoriasis patients	Inhibited the activity of the β-Catenin signaling pathway.	(69)
	LC3	Psoriasis patients	Promotion of LC3 protein.	(201)
	HMGB1	C57BL/6 mice	Basic crosstalk between HMGB1- associated autocrine and γδT cells.	(203)
	Matrine	Female BALB/c mice HaCaT cell	Inhibition of the PI3K/Akt/mTOR signalling pathway.	(97)
	LncRNA MEG3	HaCaT cell Female BALB/c mice	Inhibition of the PI3K/Akt/mTOR signalling pathway.	(98)
	PSORI-CM02	Male BALB/c mice HaCaT cell	Inhibition of the PI3K/Akt/mTOR signalling pathway.	(99)
	YXJD	Male BALB/c mice HaCaT cell	Inhibition of the PI3K/Akt/mTOR signalling pathway.	(100)
	Rapamycin	Female C57BL/6 mice	Inhibition of the PI3K/Akt/mTOR signalling pathway.	(101)
SLE	MiR-125b	SLE patients	Increased UVRAG expression and autophagy activity.	(115)
	Chloroquine	Female MRL/MpJ-Faslpr/J mice, SLE patients	Autophagy inhibition balances Th17/Treg-mediated immune	(116)
	P140	MRL/lpr and CBA/J mice	Altered autophagy processes in MRL/lpr B cells.	(117)
	Vitamin D	HPC LN patients	Reducing abnormal autophagy to protect podocytes from damage.	(121)
	Inhibition of Notch-Hes-1	Female MRL/lpr mice Female C57BL/6 mice	Inhibition of macrophage autophagic death.	(120)
	SGLT2 inhibitors	MRL/lpr mice	Reduced inflammation and enhanced autophagy.	(122)
	HNK	Female NZB/W F1 mice	Regulation of sirtuin 1 autophagy axis.	(123)
Vitiligo	Calcipotriol	Melanocyte	Protects melanocytes from oxidative damage in vitiligo.	(134)
	Anti-TRPM2	Keratinocyte	Promoting the coupling of Atg12 and Atg5.	(137)
	ATG7	Melanocyte	Protection of melanocytes against oxidative stress-induced apoptosis.	(213)
	LC3	MNT-1 cell	Promotion of tyrosinase expression.	(212)
	LBP	Melanocyte	Activation of the Nrf2/p62 signalling pathway.	(135)
	HSF1-ATG5/12	Melanocyte Vitiligo patients	Autophagy activation aids melanocyte survival under oxidative stress.	(133)
AD	CEBPB	Atg5WT mice Atg7WT mice SQSTM1WT mice Lyz2-cre mice MRP8-cre mice	Upregulation of SOCS1 and SOCS3 expression.	(160)
	WLJP-025p	SPF female BALB/c mice	Up-regulation of p62 activates Nrf2 and promotes ubiquitination and degradation of NLRP3.	(152)
	IL-37b	C57BL/6 mice	Regulates the AMPK-mTOR signalling pathway.	(153)
	Moisturizer	AD patients	Stimulate autophagy.	(150)
	Dictamnine	Male BABL/c mice	Increased LC3 expression in macrophages.	(149)
	β-Defensin-3	C57BL/6 mice	Promotion of LC3 expression and inhibition of p62 expression.	(148)
AA	PINK1	ORS cell	Mitochondrial autophagy factors that inhibit inflammatory vesicle activation.	(169)
	Dietary protein	AA patients	Prevention of autophagy disorders.	(179)
SSc	2-ME	SSc patients Fibroblast cell	Reduction of CTGF and collagen I production through PI3K/Akt/mTOR/HIF-1α signalling pathway.	(199)
	miR-126-3p	HUVEC C57BL/6 mice	Regulates the SLC7A5/mTOR signalling pathway.	(192)
	Anti-TGFβ	SSc patients Fibroblast cell	Upregulates MYST1 expression.	(186)

AA, Alopecia areata; AD, Atopic dermatitis; Akt, Protein kinase B; ATG7, Autophagy-related proteins; CEBPB, CCAAT/enhancer binding protein beta; HMGB1, High mobility group box-1 protein; HNK, Honokiol; HUVEC, Human umbilical vein endothelial cells; LC3, Microtubule-associated protein 1 light chain 3; LBP, Lipopolysaccharide-binding protein; MEG3, Maternally expressed gene 3; MIR-126, MicroRNAs-126; PIK3, Phosphatidylinositol-3-kinase; PINK1, PTEN-induced putative kinase 1; SLE, Systemic lupus erythematosus; SSc, Systemic sclerosis; SOCS, Suppressor of cytokine signaling; TRPM2, Transient receptor potential M2 channels; TGFβ, Transforming growth factor beta; UVRAG, UV radiation resistance associated gene; YXJD, Yangxue Jiedu Fang; 2-ME, 2-Methoxyestradiol.

Accumulation of the autophagy-associated protein p62 has also been found in psoriasis, which can lead to keratotic insufficiency in psoriasis, suggesting that targeting and reducing p62 expression is an effective means of treating psoriasis (205). Cimicifugae Rhizoma - Smilax glabra Roxb (CS), the main ingredient of the traditional Chinese medicine formula Shengma Xieyu Tang, which can alleviate inflammation in psoriasis by targeting and inhibiting the MAPK autophagy-related signaling pathway (206). Salvia miltiorrhiza, a traditional Chinese medicine, is the dried root and rhizome of Salvia miltiorrhiza, family Labiatae, which can alleviate the symptoms of psoriasis by targeting and inhibiting MAPK and NF-κB autophagy-related pathways, thus achieving the treatment of psoriasis (207). Paeoniflorin, the main active ingredient of the Chinese medicine Paeonia lactiflora, can exert anti-psoriasis effects by targeting and inhibiting the phosphorylation of the MAPK autophagy-related pathway (208).

One study found that increased expression of autophagy-related proteins ATG5 and ATG16L2 promotes the development of SLE, suggesting that targeting and reducing the expression of ATG5 and ATG16L2 can treat SLE (107). Dihydromyricetin is a dihydroflavonol flavonoid compound extracted from the traditional Chinese medicine Ampelopsis megalophyllaDiels et Gilg, which alleviates the symptoms of SLE by targeting inhibition of the mTOR autophagy-related signaling pathway and promoting the expression of LC3-II and Beclin-1 autophagy-related genes, thereby treating SLE (209).

Ranunculin is an epoxy diterpene lactone compound extracted from the roots, leaves, flowers, and fruits of Ranunculus officinalis, which reduces inflammation and ameliorates SLE symptoms by targeting and inhibiting the expression of autophagy-associated proteins LC3II/I and P62 (209). Artemisinin, an active ingredient extracted from the traditional Chinese medicine Artemisia annua, is a sesquiterpene lactone, which exerts anti-SLE inflammatory effects by targeting and up-regulating the expression levels of LC3-II and ATG5 autophagy-related proteins (210).

Vitiligo is characterized by a reduction in melanin due to destruction of melanocytes, and the autophagy-related proteins ATG7, ATG4, LC3 and Beclin1 contribute to melanin production (211). Increased expression of LC3 enhances melanin synthesis (212), Increased expression of ATG7 protects melanocytes from oxidative stress damage (213), ATG4 was previously mentioned as an autophagy-associated protein that promotes LC3 expression. These studies suggest that targeting increased expression of ATG7, ATG4, LC3, and Beclin1 can treat vitiligo.

San Bai Tang is a traditional Chinese herbal formula whose main ingredients include Atractylodes macrocephala, Paeonia lactiflora, and Poria, among other herbs, and which can treat vitiligo by targeting inhibition of the MAPK autophagy-related pathway (214). MA 128 is an herbal formula developed by a Korean team and consists of a number of traditional anti-allergy and anti-inflammatory herbs. It treats vitiligo by targeting and inhibiting the MAPK autophagy-related pathway to relieve the symptoms of vitiligo (215). The traditional Chinese medicine tonic acid is derived from the fruit of the leguminous plant tonic acid, which can treat vitiligo by targeting and inhibiting MAPK and NF-κB autophagy-related pathways (216).

Myricetin, a flavonol compound, is an active ingredient extracted from the stem and leaves of Garcinia Cambogia or from the bark and leaves of the Yangmei tree, which can exert anti-AD effects by targeting inhibition of the NF-κB autophagy-related signaling pathway (217). Compound traditional Chinese medicine dermatitis ointment (CTCMDO) consists of an oil extract of five herbals (Rhizoma Coptidis, Phellodendron Bark, Angelica Sinensis, Radix et Rhizoma Dioscoreae and Curcuma longa). CTCMDO can effectively improve AD by targeting and inhibiting the MAPK autophagy-related signaling pathway (218). Ren et al. found that a combination of traditional Chinese herbs, Saposhnikoviae radix, astragali radix and xidium monnieri, could treat AD by targeting inhibition of the MAPK and JAK autophagy-related signaling pathways (219).

Reduced expression of autophagy-related proteins ATG5 and LC3B in the hair matrix was found in patients with AA, suggesting that autophagy is impaired in AA, suggesting that targeting to increase the expression of ATG5 and LC3B could be helpful in the treatment of AA (167). However, it has been shown that ATG5 and LC3B expression is also elevated in AA patients, presumably as a compensatory mechanism for impaired autophagy (220).

Astragaloside IV is a saponin analogue from the traditional Chinese medicine Astragalus membranaceus, which can treat AA by targeting and inhibiting the MAPK autophagy-related signaling pathway (221). The Chinese medicine crataegus pinnatifida is derived from the dried mature fruit of Crataegus pinnatifida, family Rosaceae, which can alleviate AA symptoms by targeting the activation of MAPK and Akt autophagy-related signaling pathways, thus achieving the purpose of treating AA (222). Icariin is the main active ingredient of the Chinese medicine Epimedium, which can treat AA by targeting the activation of the PI3K/Akt autophagy-related signaling pathway (223).

Salvianolic acid B (SAB) is a bioactive ingredient extracted from Salvia miltiorrhiza, which can be used to treat SSc by attenuating dermal fibrosis by targeting inhibition of the MAPK autophagy-related signaling pathway (224). Icariin (ICA), a flavonoid glycoside extracted from the traditional Chinese medicine Epimedium, can play a therapeutic role in the treatment of SSc by alleviating the symptoms of SSc through the targeted inhibition of the JNK/NF-κB autophagy-related signaling pathway (225). Lignans (an herbal ingredient) are natural flavonoids that treat SSc by targeting and inhibiting the NF-κB autophagy-related signaling pathway to reduce the inflammatory response in SSc (226).

## 6 Conclusion

Autophagy is an intracellular self-digestive process by which cells can break down and recycle damaged organelles, abnormal protein aggregates, and other molecules within them to maintain normal cellular function and survival. There are three types of autophagy: macrophage, chaperone-mediated autophagy, and microphagy, each with a different molecular mechanism. Autophagy plays different roles in different skin cells, but all serve to maintain skin cell homeostasis. Dysregulation of autophagy leads to various skin diseases such as psoriasis, SLE, vitiligo, AD, AA and SSc, but autophagy is also a potential target for the treatment of these diseases.

Notably, PSORI-CM02, YXJD, Picrasidine, HNK, Azadirachtin, LBP, Cortex Dictamni, and WLJP-025p, which are the herbal formulations or herbal ingredients mentioned in the above article, can be used to treat the corresponding diseases by modulating autophagy, suggesting that there exists a large number of potential herbal-autophagy-targeted therapeutic approaches that are worth exploring in the future.
